# Polyunsaturated Fatty Acids Modulate the Association between *PIK3CA-KCNMB3* Genetic Variants and Insulin Resistance

**DOI:** 10.1371/journal.pone.0067394

**Published:** 2013-06-27

**Authors:** Ju-Sheng Zheng, Donna K. Arnett, Laurence D. Parnell, Yu-Chi Lee, Yiyi Ma, Caren E. Smith, Kris Richardson, Duo Li, Ingrid B. Borecki, Katherine L. Tucker, José M. Ordovás, Chao-Qiang Lai

**Affiliations:** 1 Department of Food Science and Nutrition, Zhejiang University, Hangzhou, China; 2 Jean Mayer USDA Human Nutrition Research Center on Aging at Tufts University, Boston, Massachusetts, United States of America; 3 Department of Epidemiology, University of Alabama at Birmingham, Birmingham, Alabama, United States of America; 4 Department of Genetics, Washington University School of Medicine, Saint Louis, Missouri, United States of America; 5 Department of Health Sciences, Northeastern University, Boston, Massachusetts, United States of America; Sapienza University of Rome, Italy

## Abstract

**Background:**

Neighboring genes *PIK3CA* and *KCNMB3* are both important for insulin signaling and β-cell function, but their associations with glucose-related traits are unclear.

**Objective:**

The objective was to examine associations of *PIK3CA-KCNMB3* variants with glucose-related traits and potential interaction with dietary fat.

**Design:**

We first investigated genetic associations and their modulation by dietary fat in the Genetics of Lipid Lowering Drugs and Diet Network (GOLDN) study (n = 820). Nine single-nucleotide polymorphisms (SNPs) were selected for analysis, covering more than 80% of the SNPs in the region. We then sought to replicate the findings in the Boston Puerto Rican Health Study (BPRHS) (n = 844).

**Results:**

For *KCNMB3* missense mutation rs7645550, meta-analysis indicated that homeostasis model assessment of insulin resistance (HOMA-IR) was significantly lower in minor allele T homozygotes compared with major allele C carriers (pooled *P*-value = 0.004); for another SNP rs1183319, which is in moderate LD with rs7645550, minor allele G carriers had higher HOMA-IR compared with non-carriers in both populations (pooled *P*-value = 0.028). In GOLDN, rs7645550 T allele homozygotes had lower HOMA-IR only when dietary n-3: n-6 PUFA ratio was low (≤0.11, *P* = 0.001), but not when it was high (>0.11, *P*-interaction = 0.033). Similar interaction was observed between rs1183319 and n-3: n-6 PUFA ratio on HOMA-IR (*P*-interaction = 0.001) in GOLDN. Variance contribution analyses in GOLDN confirmed the genetic association and gene-diet interaction. In BPRHS, dietary n-3: n-6 PUFA ratio significantly modulated the association between rs1183319 and HbA1c (*P*-interaction = 0.034).

**Conclusion:**

*PIK3CA-KCNMB3* variants are associated with insulin resistance in populations of different ancestries, and are modified by dietary PUFA.

## Introduction

Type 2 diabetes (T2D), one of the most common chronic diseases in the world, is predicted to affect 285 million adults in 2010 and 439 million adults by 2030 [1]. Insulin resistance (IR) is central to the development of T2D, and usually precedes diagnosis of T2D by many years. Observational and intervention studies suggest that IR could be prevented by dietary and lifestyle modifications [2], including quantity and quality of dietary fat [3]. However, the beneficial effect of n-3 polyunsaturated fatty acid (n-3 PUFA) on the prevention of IR and T2D is still controversial [4]–[13]. A possible reason for this discrepancy is the fact that genetic factors alone or through interactions with diets and lifestyle influence the pathogenesis of IR and T2D [14]–[16]. Although numbers of genetic variants associated with IR continue to grow, relatively few studies have examined the interactions of genetic variants with fatty acid intake for IR. One of the novel candidate genes is *PIK3CA*, encoding phosphatidylinositol 3-kinase (PI3K) Class-IA p110α catalytic subunit. PI3K is a key mediator of insulin signaling and implicated in human diseases including diabetes and cancer [17]. PI3K is activated by the tyrosine-phosphorylated insulin receptor substrate following insulin stimulation, and subsequently catalyzing the formation of phosphatidylinositol (3,4,5)-trisphosphate, thereby initiating downstream pathways, leading to glucose uptake and glycogen synthesis [17], [18]. PI3K Class-IA p110α catalytic subunit is the primary insulin-responsive PI3K isoform [19], [20] and is important in growth factor and metabolic signaling [20], [21]. Mice with hepatic knockout of *Pik3ca* showed reduced insulin sensitivity, impaired glucose tolerance, and increased gluconeogenesis [20]. However, the relationships of *PIK3CA* mutations with IR in humans and whether any *PIK3CA*-nutrient interaction modulates IR are still unclear. The *KCNMB3* gene, encoding potassium large conductance calcium-activated channel (BK channel) M beta member 3, is 5 kbp downstream of *PIK3CA* and is in a large linkage disequilibrium (LD) block with this gene. BK channels are a type of “large conductance” channel that transports potassium ions (K^+^) through cell membranes. As regulators of glucose homeostasis, these channels are important for β-cell function and regulate insulin secretion [22], [23]. To our knowledge, no studies have reported the association of *KCNBM3* variants with IR and the potential interactions of these variants with dietary fatty acids.

The aim of the present study was to examine the associations between variants across the *PIK3CA-KCNMB3* region and IR and other glucose-related traits with potential modifications by dietary fat in a non-Hispanic white population in the Genetics of Lipid Lowering Drugs and Diet Network (GOLDN) study [24]. Replication of the observed relationships was evaluated in an independent population of Puerto Rican adults living in Massachusetts and participating in the Boston Puerto Rican Health Study (BPRHS) [25].

## Methods and Materials

### Ethics Statement

For GOLDN, the protocol was approved by the Institutional Review Boards at the University of Alabama, the University of Minnesota, the University of Utah and Tufts University. For the BPRHS, the study protocol was approved by the Institutional Review Boards at Tufts University and Northeastern University. All the participants gave informed consent.

### Study Populations

The GOLDN study (n = 820) consisted of 406 men and 414 women. All participants were of European origin, recruited from two National Heart, Lung, and Blood Institute Family Heart Study field centers (Minneapolis, MN, and Salt Lake City, UT) [26]. The study design and methodology were described previously [24]. Subjects were asked to take the lipid lowering drug (micronized fenofibrate, 160 mg) in a three-week unblinded clinical trial. The registration identifier is NCT00083369 (http://www.clinicaltrials.gov/ct2/show/NCT00083369). Clinical and biochemical parameters were collected before and after the intervention, and all the fasting blood biochemical parameters used in the present study were from the second visit before the intervention.

The replication study included 844 participants (239 men, 605 women) from the BPRHS, with almost complete genotype and dietary assessment data. The study details were described in detail previously [25]. Briefly, the BPRHS is a longitudinal cohort study for stress, nutrition, health, and aging, for which participants who self-identified as Puerto Rican and lived in the Boston metropolitan area were recruited. Ancestry composition of the BPRHS is 57.2% European, 27.4% African, and 15.4% Native American [27].

### Data Collection

In GOLDN, dietary information was collected by a Diet History Questionnaire (DHQ) developed by the National Cancer Institute, based on national dietary data (USDA Continuing Survey of Food Intakes by Individuals). The DHQ has been validated in two studies [28], [29]. In the BPRHS, dietary intake was assessed by a validated Food Frequency Questionnaire (FFQ) designed for and tested in this population [30], and nutrient analyses were linked to the Minnesota Nutrient Data system (1999, version 25). Importantly, PUFA intakes have been validated for both populations [27], [28], [29] and measures of erythrocyte membrane PUFA content in GOLDN is significantly correlated with dietary PUFA (r = 0.11, P = 0.002). Lifestyle and demographic information, medication use and medical history were obtained in each population by questionnaire. Anthropometric measurements were taken at the baseline visit. Height, body weight and waist circumference were all measured by standard techniques. Body mass index was calculated as weight (kg)/height (m)^2^. T2D was defined based on a fasting plasma glucose concentration ≥126 mg/dL or use of insulin or other diabetes medication.

### Laboratory Methods

Blood samples were drawn after an overnight fast. In GOLDN, fasting insulin was measured by a commercial kit using a radioimmunoassay (Linco Research, St. Charles, MO), fasting glucose was determined by a hexokinase-mediated reaction on the Hitachi commercial kit (Linco Research, St. Charles, MO). In the BPRHS, measurements of fasting insulin, glucose and glycosylated hemoglobin (HbA1c) have been described in details previously [25]. Homeostasis model assessment of IR (HOMA, fasting glucose×fasting insulin/22.5) was used to assess IR in both populations.

### Single-nucleotide Polymorphism Selection, Genotyping and Haplotype Analysis

Nine tag single-nucleotide polymorphisms (SNP) were selected using Tagger [31] based on HapMap Caucasian European Utah data (www.hapmap.org) in the Haploview program (version 4.2) with a minor allele frequency ≥0.05 and r^2^>0.80. The selected SNPs covered more than 80% of all SNPs in the region (160 kbp), ranging from 50 kbp upstream of the *PIK3CA* gene to 25 kbp downstream of the gene and covering *KCNMB3*. LD of the selected SNPs were estimated as correlation coefficients (r^2^) using Haploview (**Figure S1**). Haplotype of these SNPs were also estimated by Haploview.

DNA was isolated from blood samples using Gentra Puregene Blood Kits (Gentra Systems) in GOLDN and QIAamp DNA Blood Mini Kits (Qiagen) in the BPRHS. In GOLDN, genome-wide genotyping was conducted by using Affymetrix Genome-Wide Human SNP Array 6.0. MACH (Markov Chain based haplotyper V1.0.16, http://www.sph.umich.edu/csg/abecasis/MaCH/) was used to impute all autosomal SNPs using the phased haplotypes for CEU from HapMap (release 22, build 36) as a reference panel. In the BPRHS, genome-wide genotyping was conducted by using Illumina HumanOmni5-Quad GWAS arrays.

### Selection of SNPs for Replication in the BRPHS and Population Admixture

Two SNPs: rs7645550 and rs1183319 were selected for replication in BPRHS based on evidence of significant relationships in GOLDN. One SNP, rs7645550, is a missense mutation in the *KCNMB3* gene. The second SNP, rs1183319 is located in the 5′UTR of *KCNMB3* gene. In the BPRHS population, the population admixture was estimated previously based on 100 SNPs that were selected as ancestry informative [27]. All the genotype-associated analyses were adjusted for the population admixture in the present study.

### Statistical Analyses

Statistical analyses were performed using SAS (version 9.2) and R software (version 2.15.0). HOMA-IR, insulin, glucose and HbA1c were Box-Cox transformed [32] to achieve normal distribution prior to statistical analysis. Chi-square tests were conducted to examine whether the selected SNPs were in Hardy-Weinberg equilibrium. Due to the limited sample size, a dominant model was applied in the final analysis to increase statistical power for all SNPs, except for rs7645550, which followed a clear recessive model for HOMA-IR (HOMA-IR = 3.18, 3.67, and 3.71 for TT, CT and CC carriers, respectively). The minor allele was always treated as the reference allele in the models. The correlations of dietary fatty acids with glucose-related traits were assessed using a Pearson correlation coefficient after controlling for potential confounders. Dietary total fat, saturated fatty acid (SFA), monounsaturated fatty acids (MUFA), total PUFA, n-3 PUFA, n-6 PUFA and n-3: n-6 PUFA ratio were dichotomized based on the median intake of each population for the gene-nutrient interaction analysis. In GOLDN, the family relationships within the population were adjusted using the SAS GENMOD procedure, assuming an exchangeable correlation structure within pedigree. A multivariate interaction model was used to test the interactions of fatty acid intake with genetic variants after adjustment for age, sex, waist circumference, alcohol drinking, smoking status, physical activity, T2D and study center in the GOLDN population. In the BPRHS, using linear regression model, similar genotype associations and interactions were tested for selected SNPs while adjusting for age, sex, waist circumference, alcohol drinking, smoking status, physical activity, T2D and population admixture. Variance contribution of the genetic effect and the gene-nutrient interaction for HOMA-IR were estimated using a GWAF package [33] in R.

Meta-analysis was conducted with the Meta-Analysis Helper (METAL) (http://www.sph.umich.edu/csg/abecasis/metal/) under fixed-effects models, and combining the z-statistics across the two populations, weighted by sample size. Meta-analysis was only conducted to summarize the results from main genetic association, but not for gene-nutrient interaction, as the dietary intake range and FFQ used are substantially different between the two populations. To correct for multiple testing, Bonferroni adjustment was used. As our primary interest is insulin resistance, we only applied Bonferroni correction for HOMA-IR. The pertinent number of correction due to multiple testing is 63 (7×9), and the corrected *P*-value for a significant interaction is 0.0008 (0.05/63).

## Results

### Characteristics of the Populations and Genetic Variants at *PIK3CA-KCNMB3*

Men had significantly higher total energy and MUFA intake than women in both GOLDN and the BPRHS populations (**Table 1**). In GOLDN, none of the nine SNPs deviated from Hardy-Weinberg equilibrium expectations (*P*>0.01), with the minor allele frequencies ranging from 0.07 to 0.49 (**Table S1**). In the BPRHS, the minor allele frequency of rs7645550 (T) and rs1183319 (G) was 0.36 and 0.45, respectively, and did not deviate from Hardy-Weinberg equilibrium expectations (*P*>0.01). SNPs rs7645550 and rs1183319 were in moderate LD in both GOLDN (*r* ^2^ = 0.54) and BPRHS (*r* ^2^ = 0.44). Three haplotypes were observed for this pair of SNPs: CG, TA, and CA, and the corresponding frequency was 0.45, 0.40 and 0.15 in GOLDN, and 0.45, 0.36 and 0.20 in BPRHS.

**Table 1:** Characteristics of participants in GOLDN and the BPRHS^1^.

Characteristics	GOLDN		BPRHS	
	Men (n = 406)	Women (n = 414)	Men (n = 239)	Women (n = 605)
Age, y	48.8±15.9	49.0±16.1	57.6±7.7	58.1±7.1
BMI, kg/m^2^	28.6±4.7	28.4±6.2	29.9±5.1	33.0±7.0*
Waist circumference, cm	101±14	93.4±17.5*	102±14	102±16
Current smoker, n (%)	33 (8.1)	34 (8.2)	70 (29.9)	112 (18.7)*
Current drinker, n (%)	199 (49)	208 (50.2)	121 (50.6)	196 (32.4)*
Type 2 diabetes, n (%)	33 (8.1)	26 (6.3)	106 (44.4)	254 (42.0)
Energy intake, kJ/d	10481±6282	7450±3419*	12134±6353	9200±4874*
Total fat intake, %	35.9±6.7	35.1±6.9	32.4±6.0	31.4±5.5
Saturated fat intake, %	12.1±2.7	11.6±2.6*	9.8±2.7	9.4±2.3
MUFA intake, %	13.6±2.8	13.0±2.8*	11.3±2.2	10.8±2.1*
PUFA intake, %	7.4±2.0	8.0±2.3*	8.8±2.1	8.6±2.1
n-3 PUFA intake, %	0.68±0.19	0.75±0.23*	0.69±0.18	0.71±0.19
n-6 PUFA intake, %	6.64±1.83	7.14±2.16*	8.07±1.99	7.91±2.00
n-3: n-6	0.10±0.02	0.11±0.02	0.09±0.02	0.09±0.03
HOMA-IR	3.87±2.63	3.37±2.31*	5.95±7.69	5.80±7.16
Glucose, mmol/L	5.87±1.19	5.46±0.94*	6.93±2.91	6.77±2.83
Insulin, pmol/L	101±58	94.5±56.5	124±117	125±113
HbA1c, %	NA	NA	7.09±1.96	7.11±1.78

1 Values are mean ± SD, or n (%).

* *P*<0.01 different from men within the population; HbA1c, glycosylated hemoglobin; NA, not available.

### Associations between *PIK3CA-KCNMB3* Variants and Glucose-related Traits

Meta-analysis indicated that SNP rs7645550 minor allele T homozygotes had significantly lower HOMA-IR (z-score = 2.86, pooled *P*-value = 0.004), insulin (z-score = 2.33, pooled *P*-value = 0.02) and glucose concentration (z-score = 2.83, pooled *P*-value = 0.005) compared with major allele C carriers (**Table 2**). For SNP rs1183319, minor allele G carriers had significantly higher HOMA-IR (z-score = 2.19, pooled *P*-value = 0.028) and glucose concentration (z-score = 3.14, pooled *P*-value = 0.002) compared with major allele A homozygotes.

**Table 2:** Associations between *PIK3CA-KCNMB3* variants and glucose-related traits in the GOLDN and BPRHS participants^1^.

	Genotypes (n)	GOLDN		BPRHS		Meta-analysis		
		Mean ± SEM (n)	*P*-value	Mean ± SEM (n)	*P*-value	Z-score	*P*-value	*I^2^* (%)
**HOMA-IR**								
rs7645550	CC+CT	3.69±0.10 (698)	0.004	5.93±0.28 (730)	0.234	2.86	0.004	31.6
	TT	3.18±0.19 (122)		5.33±0.62 (113)				
rs1183319	AG+GG	3.66±0.10 (571)	0.060	5.97±0.29 (680)	0.240	2.19	0.028	0
	AA	3.50±0.16 (249)		5.41±0.47 (159)				
**Insulin**								
rs7645550	CC+CT	99.4±2.2 (698)	0.048	126±4 (730)	0.185	2.33	0.02	0
	TT	88.5±4.5 (122)		117±11 (113)				
rs1183319	AG+GG	98.5±2.4 (571)	0.319	127±5 (680)	0.483	1.20	0.229	0
	AA	96.0±3.7(249)		118±8 (159)				
**Glucose**								
rs7645550	CC+CT	5.70±0.04 (698)	0.0001	6.80±0.11 (730)	0.895	2.83	0.005	86.1
	TT	5.46±0.09 (122)		6.90±0.27 (113)				
rs1183319	AG+GG	5.68±0.04 (571)	0.008	6.84±0.11 (680)	0.120	3.14	0.002	0
	AA	5.62±0.08 (249)		6.73±0.21 (159)				

1 Values are mean ± SEM. *P*-values were adjusted for age, sex, waist circumference, alcohol drinking, smoking status, physical activity, type 2 diabetes, study center and family relationships in GOLDN; *P*-values were adjusted for age, sex, waist circumference, alcohol drinking, smoking status, physical activity, type 2 diabetes and population admixture in BPRHS. Meta-analysis was used to combine Z statistics across GOLDN and the BPRHS, weighted by the sample size; I^2^ was used to assess statistical heterogeneity: I^2^ values of 25%, 50% and 75% correspond to cut-off points for low, moderate and high degrees of heterogeneity.

### Associations between Dietary Fatty Acids and Glucose-related Traits

In GOLDN, the ratio of dietary n-3: n-6 PUFA was positively correlated with the fasting glucose concentration (r = 0.081, *P* = 0.023) after controlling for age, sex, waist circumference, alcohol drinking, smoking status, physical activity, T2D and study center. In the BPRHS, this ratio was inversely correlated with HOMA-IR (r = −0.069, *P* = 0.05) and insulin (r = −0.073, *P* = 0.039), and dietary n-3 PUFA was inversely correlated with insulin (r = −0.072, *P* = 0.042) after controlling for age, sex, waist circumference, alcohol drinking, smoking status, physical activity, T2D and population admixture. Other dietary fatty acid intakes, such as total fat, SFA, MUFA, PUFA, n-3 PUFA, and n-6 PUFA, were not correlated with glucose-related traits in the GOLDN or in the BPRHS population.

### Interactions of *PIK3CA-KCNMB3* Variants with Dietary Fatty Acids on Fasting Glucose and HbA1c

Genotype by diet interactions between *PIK3CA-KCNMB3* variants and dietary total fat, SFA, MUFA, PUFA, n-3 PUFA, n-6 PUFA and n-3: n-6 PUFA ratio for glucose-related traits were examined. Dietary fatty acids were expressed as percentage of total energy intake and dichotomized into categorical variables based on the median intake of the population. In GOLDN, SNP rs1183319 had significant and marginally significant interactions with dietary MUFA (*P* = 0.014) and PUFA (*P* = 0.051) for fasting glucose (data not shown). Other genetic variants did not interact with fatty acid intakes for glucose concentration.

In BPRHS, dietary n-3: n-6 PUFA ratio significantly modulated the association between rs1183319 and HbA1c (*P* = 0.034) (**Table S2**). The G allele carriers had higher HbA1c compared with A homozygotes when the n-3: n-6 ratio was high (>0.09, *P* = 0.011), but not when the ratio was low (≤0.09, *P* = 0.635).

### Interactions of *PIK3CA-KCNMB3* Variants with Dietary Fatty Acids for HOMA-IR and Insulin

**Figure 1:**
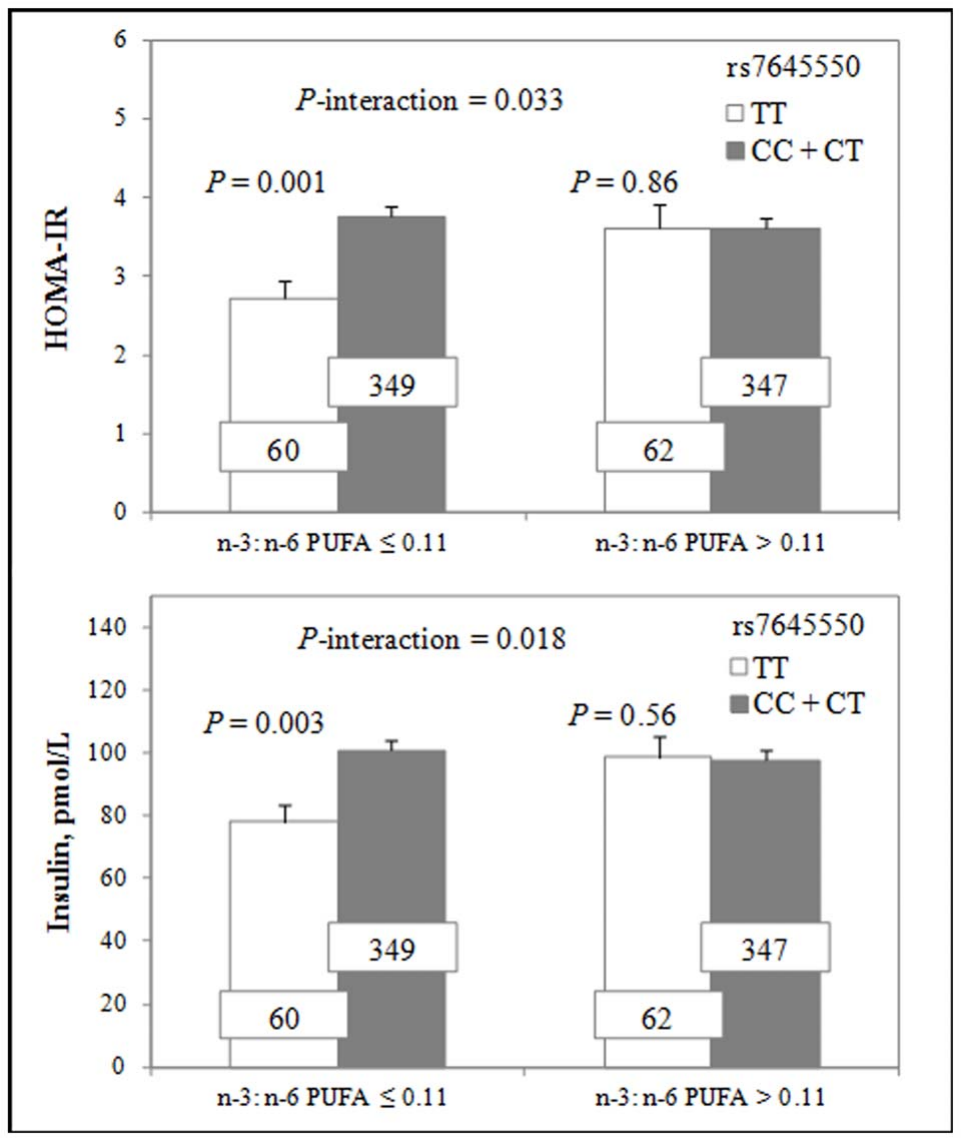
Interactions between rs7645550 and dietary n-3: n-6 PUFA ratio on HOMA-IR and insulin in GOLDN. When dietary n-3: n-6 PUFA ratio was low (≤0.11), there were significant differences by genotypes for HOMA-IR (*P* = 0.001) and insulin (*P* = 0.003), while no significant differences for HOMA-IR (*P* = 0.86) and insulin (*P* = 0.56) were observed among the rs7645550 genotypes when dietary n-3: n-6 PUFA ratio was high (>0.11). The number inside the bar indicates the number of participants with a given genotype.

In GOLDN, significant interactions between dietary PUFA and *PIK3CA-KCNMB3* variants for HOMA-IR were observed (**Table 3** **,** **Figure 1**). SNPs rs7645550 and rs1183319 significantly interacted with the n-3: n-6 ratio to modulate HOMA-IR (*P* = 0.033 for rs7645550, *P* = 0.001 for rs1183319) and insulin (*P* = 0.018 for rs7645550, *P* = 0.004 for rs1183319). SNP rs7645550 minor allele T homozygotes had lower HOMA-IR than C allele carriers (*P* = 0.001) only when the n-3: n-6 ratio was low (≤0.11) (**Figure 1**). Minor allele G carriers of rs1183319 had significantly higher HOMA-IR than non-carriers when the dietary n-3: n-6 PUFA ratio was low (≤0.11, *P*<0.001), and not when the ratio was high (>0.11, *P* = 0.368) (**Table 3**).

**Table 3:** Interactions between dietary fatty acids and *PIK3CA-KCNMB3* variants on HOMA-IR in the GOLDN participants^1^.

Dietaryfatty acids	% energy	rs1183319				rs7642066			
		AG+GG (n)	AA (n)	*P*-trend	*P* –interaction^2^	AA+AT (n)	TT (n)	*P* -trend	*P* -interaction^2^
Total fat	≤35.66	3.40±0.12 (289)	3.60±0.22 (120)	0.467	0.003	3.52±0.13 (287)	3.32±0.16 (122)	0.278	0.045
	>35.66	3.94±0.17 (281)	3.41±0.22 (128)	<0.001		3.62±0.15 (282)	4.13±0.30 (127)	0.105	
Total MUFA	≤13.23	3.49±0.15 (278)	3.57±0.21 (131)	0.601	0.009	3.52±0.14 (287)	3.52±0.25 (122)	0.097	0.002
	>13.23	3.83±0.15 (292)	3.43±0.24 (117)	0.002		3.62±0.15 (282)	3.93±0.23 (127)	0.013	
Total SFA	≤11.82	3.58±0.14 (290)	3.54±0.23 (119)	0.957	0.024	3.52±0.14 (285)	3.67±0.22 (124)	0.951	0.855
	>11.82	3.76±0.16 (280)	3.47±0.22 (129)	0.003		3.62±0.14 (284)	3.77±0.27 (125)	0.785	
Total n-3 PUFA	≤0.68	3.49±0.13 (289)	3.37±0.19 (120)	0.293	0.588	3.56±0.13 (286)	3.21±0.17 (123)	0.227	0.059
	>0.68	3.85±0.17 (281)	3.62±0.25 (128)	0.063		3.57±0.15 (283)	4.24±0.29 (126)	0.115	
Total n-6 PUFA	≤6.60	3.55±0.12 (280)	3.42±0.17 (129)	0.498	0.197	3.61±0.12 (292)	3.27±0.18 (117)	0.129	0.008
	>6.60	3.78±0.17 (290)	3.60±0.27 (119)	0.015		3.53±0.16 (277)	4.15±0.28 (132)	0.018	
n-3: n-6	≤0.11	3.81±0.16 (299)	3.05±0.17 (110)	<0.001	0.001	3.46±0.13 (272)	3.90±0.27 (137)	0.040	0.018
	>0.11	3.50±0.13 (271)	3.86±0.25 (138)	0.368		3.67±0.15 (297)	3.51±0.19 (112)	0.178	
Total PUFA	≤7.34	3.57±0.13 (282)	3.42±0.18 (127)	0.466	0.240	3.63±0.12 (293)	3.26±0.18 (293)	0.119	0.008
	>7.34	3.76±0.17 (288)	3.59±0.27 (121)	0.022		3.50±0.16 (276)	4.14±0.28 (133)	0.018	

1 Values are mean ± SEM.

2 *P*-values were derived from a multivariate interaction model, after adjustment for age, sex, waist circumference, alcohol drinking, smoking status, physical activity, type 2 diabetes, study center and family relationships.

In addition to rs7645550 and rs1183319, SNP rs7642066 was found to interact significantly with dietary PUFA, MUFA, n-6 PUFA, and the n-3: n-6 ratio on HOMA-IR (**Table 3**). Furthermore, rs2677764 showed a significant interaction with dietary n-3 PUFA to modulate HOMA-IR (*P* = 0.020, data not shown) in GOLDN. No significant interactions between rs7645550 and rs118339 and dietary fat for HOMA-IR in the BPRHS were observed.

### Variance Contributions by GxE Interaction for HOMA-IR in GOLDN

Genotype by environment (GxE) interactions may contribute substantially to phenotypic variation, as the main genetic effect usually inadequately explains phenotypic variation. In order to examine the portion of phenotypic variation explained by the *PIK3CA-KCNMB3* variants and their interaction with diet, the GWAF package in R was used to examine the variance contribution in GOLDN, after controlling for potential confounders. We estimated the variance contribution by the GxE interaction of the n-3: n-6 PUFA ratio with the *PIK3CA-KCNMB3* variants to the total HOMA-IR variance, as this ratio was the most important dietary factor affecting the main genetic effect for HOMA-IR in the present study. The total portion of HOMA-IR variation explained by *PIK3CA-KCNMB3* variants (six independent SNPs, with r^2^<0.20 were selected: rs3975506, rs2677760, rs2677764, rs1170672, rs1183319, and rs7642066) was 1.91%, and that explained by the interaction of *PIK3CA-KCNMB3* variants with dietary n-3: n-6 PUFA ratio was 1.78% (**Table S3**).

## Discussion

In the present study, we found that genetic variants at the *PIK3CA-KCNMB3* region associated with glucose-related traits, and had significant interactions with dietary PUFA to modulate these traits, especially HOMA-IR. SNP rs7645550 minor allele T homozygous carriers had lower HOMA-IR compared with major allele C carriers in both European-American (GOLDN) and Puerto Rican (BPRHS) populations. For rs1183319, minor allele G carriers had higher HOMA-IR compared with non-carriers in both populations. In addition, both of these two SNPs interacted with dietary n-3: n-6 PUFA ratio on HOMA-IR in the GOLDN population.

Previous studies reporting associations between IR, T2D and n-3 PUFA intake are inconclusive. Some studies found that n-3 PUFA was inversely associated with IR and risk of T2D [6], [8], [9], while others found n-3 PUFA was positively associated with IR and risk of T2D [5], [7] or had no effect on IR or T2D [4], [13]. Recent meta-analyses of randomized controlled trials found that n-3 PUFA supplementation had no significant effects on IR or glycemic control [11], [12]. Meta-analyses based on prospective studies consistently concluded that overall fish and n-3 PUFA intake were not associated with risk of T2D [10], [34], [35]. The number of genetic variants demonstrated to contribute to IR and T2D continues to expand [36], [37] and a growing number of variants have been shown to be influenced by interactions with dietary factors [36], [38]–[42]. Genetic variants in genes including *ACSL1*, *ADIPOQ*, *ADIPOR1*, *CLOCK*, *PLIN1*, *TCF7L2*, and *LEPR* [42] were found to interact with dietary fatty acids to modulate IR. Hence, inconsistent associations between n-3 PUFA and IR or T2D may be partly attributed to the influence of gene-nutrient interaction [14]–[16]. In the present study, the associations between dietary fatty acids and IR were inconsistent across the GOLDN and BPRHS populations. Dietary n-3: n-6 PUFA ratio was inversely correlated with IR in BPRHS, but not in GOLDN; while n-3: n-6 PUFA ratio was positively correlated with glucose in GOLDN. Interactions between *PIK3CA-KCNMB3* variants and n-3: n-6 PUFA ratio may contribute to such inconsistencies. The different interaction patterns may be due to differences in fatty acid intake between the two populations. For example, the mean dietary intake of n-3: n-6 PUFA ratio (0.106±0.020) in GOLDN was higher than the mean intake of n-3: n-6 PUFA ratio (0.092±0.027) in the BPRHS. On the other hand, >40% of the participants in BPRHS had T2D and most of the T2D patients took diabetes medication, which may be an important source of the different observations between the two populations. In addition, distinction in both genetic background and culture, especially as related to food choices and preparation methods, between the BPRHS and GOLDN populations could affect the observed interactions [27].

Four *PIK3CA-KCNMB3* variants were found to interact with n-3: n-6 PUFA ratio or n-3 PUFA in the GOLDN population. The threshold for the Bonferroni correction was 0.0008, and the interaction of rs1183319 with the n-3: n-6 PUFA ratio on HOMA-IR (*P* = 0.001) marginally passed the Bonferroni correction. Furthermore, these results of gene-nutrient interactions were confirmed with variance contribution analysis, and variance contribution analysis indicated that the interaction of *PIK3CA-KCNMB3* variants with n-3: n-6 PUFA ratio contributed a similar proportion of the HOMA-IR variance as did the main genetic effects. The mechanisms through which dietary n-3 PUFA modulated the association of these variants with IR and other glucose-related traits are still unclear. It is well known that n-3 PUFA and their metabolites are natural ligands for peroxisome proliferator receptor activator (PPAR) γ, and PPAR-γ is reported to regulate insulin sensitivity and glucose homeostasis through different mechanisms [43], [44]. It is predicted by the MAPPER transcription factor binding site prediction tool (http://mapper.chip.org) that the *KCNMB3* promoter has a PPAR-γ -retinoid X receptor complex binding site, through which n-3 PUFA may influence *KCNMB3* gene expression and interact with *KCNMB3* variants to influence IR. In addition, n-3 PUFA (docosahexaenoic acid) was reported to activate vascular BK channels dependent on the cytochrome P450 epoxygenase activity [45], and this mechanism may support the interactions of n-3 PUFA with genetic variants at *KCNMB3* to influence activity of BK channels. However, more research is needed to elucidate a precise mechanism.

Despite the different genetic backgrounds of the two populations, *PIK3CA-KCNMB3* variants (rs7645550 and rs1183319) were found to be associated with glucose-related traits in both populations in the current study. Both SNPs are located in the *KCNMB3* gene: rs7645550 is a missense mutation (Ala to Thr) and rs1183319 maps to the 5′-UTR. Importantly, the missense mutation of rs7645550, which changes an alanine conserved from mammals to amphibians to a threonine is predicted by PolyPhen-2 [46] to be “probably damaging”. Previous GWAS [47] indicated a tendency of association between rs1183319 (*P* = 0.08), rs7645550 (*P* = 0.198) and HOMA-IR (http://www.magicinvestigators.org/downloads/). These non-significant associations may be due to the influence of dietary factors, as suggested by this study. It is reasonable to postulate that *KCNMB3* variants may affect IR and glucose metabolism through BK channels. *KCNMB3*, encodes BK channel subunit 3, and variants at *KCNMB3* were shown to affect BK channel activity [48]. An animal study [22] suggested that BK channel knockout (BK-KO) mice displayed markedly impaired glucose tolerance and insulin secretion through effects on β-cell function. BK channels were also reported to regulate insulin secretion in mouse pancreatic beta-cells [23]. However, no study so far has reported associations between *KCNMB3* variants and IR or other glucose-related traits. Studies in additional populations are needed to further replicate these associations. Apart from *KCNMB3*, *PIK3CA* variant (rs2677760) was also reported to be associated with IR in the GOLDN population. *PIK3CA* is critical in the insulin transduction pathway and dysfunction of *PIK3CA* may lead to IR and impaired glucose tolerance, as suggested by evidence from the hepatic *Pik3ca* knockout mice [20]. However, rs2677760 is in the intron of the *PIK3CA* gene, and its function is still unclear.

Although there were significant associations between *PIK3CA-KCNMB3* variants and glucose-related traits in both GOLDN and BPRHS populations, and *PIK3CA-KCNMB3* variants were found to interact with PUFA, our results should be interpreted cautiously. First, the moderate sample sizes in both populations limit statistical power. Second, the associations between *PIK3CA-KCNMB3* variants and glucose-related traits and the modulation by dietary fatty acids were reported for the first time in the current study. Further replication in other populations is warranted.

In conclusion, we observed that *PIK3CA-KCNMB3* variants were associated with the glucose-related traits in populations of European-American and Puerto Rican ancestry. Dietary PUFA, especially the n-3: n-6 PUFA ratio, modulated the effects of *PIK3CA-KCNMB3* variants on the glucose-related traits.

## Supporting Information

Figure S1 **Linkage disequilibrium (LD) plot across** ***PIK3CA-KCNMB3*** **region in CEU population.** The horizontal white bar depicts the 160-kb DNA segment analyzed in the sample. An LD plot is presented at the bottom of the figure: each diamond represents the magnitude of LD for the single pair of markers. The numbers under the variant name and above the plot indicates the order of the corresponding variants among all the variants in the region. The numbers inside the diamonds indicate the *r* ^2^ value.
(DOCX)

Table S1 **Description of selected variants in** ***PIK3CA-KCNMB3*** **region in GOLDN.**
(DOCX)

Table S2 **Interactions between dietary PUFA and rs1183319 on fasting glucose and HbA1c in the BPRHS participants.**
(DOCX)

Table S3 **Variance contributions of** ***PIK3CA-KCNMB3*** **variants and their interaction with dietary n-3: n-6 PUFA ratio for HOMA-IR in GOLDN.**
(DOCX)
