# Supplementary material for: Polyunsaturated Fatty Acids Modulate the Association between PIK3CA-KCNMB3 Genetic Variants and Insulin Resistance
Source: PLoS One. 2013 Jun 27;8(6):e67394. doi: 10.1371/journal.pone.0067394 (PMC3694924; doi:10.1371/journal.pone.0067394)

**Figure S1. Linkage disequilibrium (LD) plot across *PIK3CA-KCNMB3* region in CEU population.** The horizontal white bar depicts the 160-kb DNA segment analyzed in the sample. An LD plot is presented at the bottom of the figure: each diamond represents the magnitude of LD for the single pair of markers. The numbers under the variant name and above the plot indicates the order of the corresponding variants among all the variants in the region. The numbers inside the diamonds indicate the *r*^2^ value.


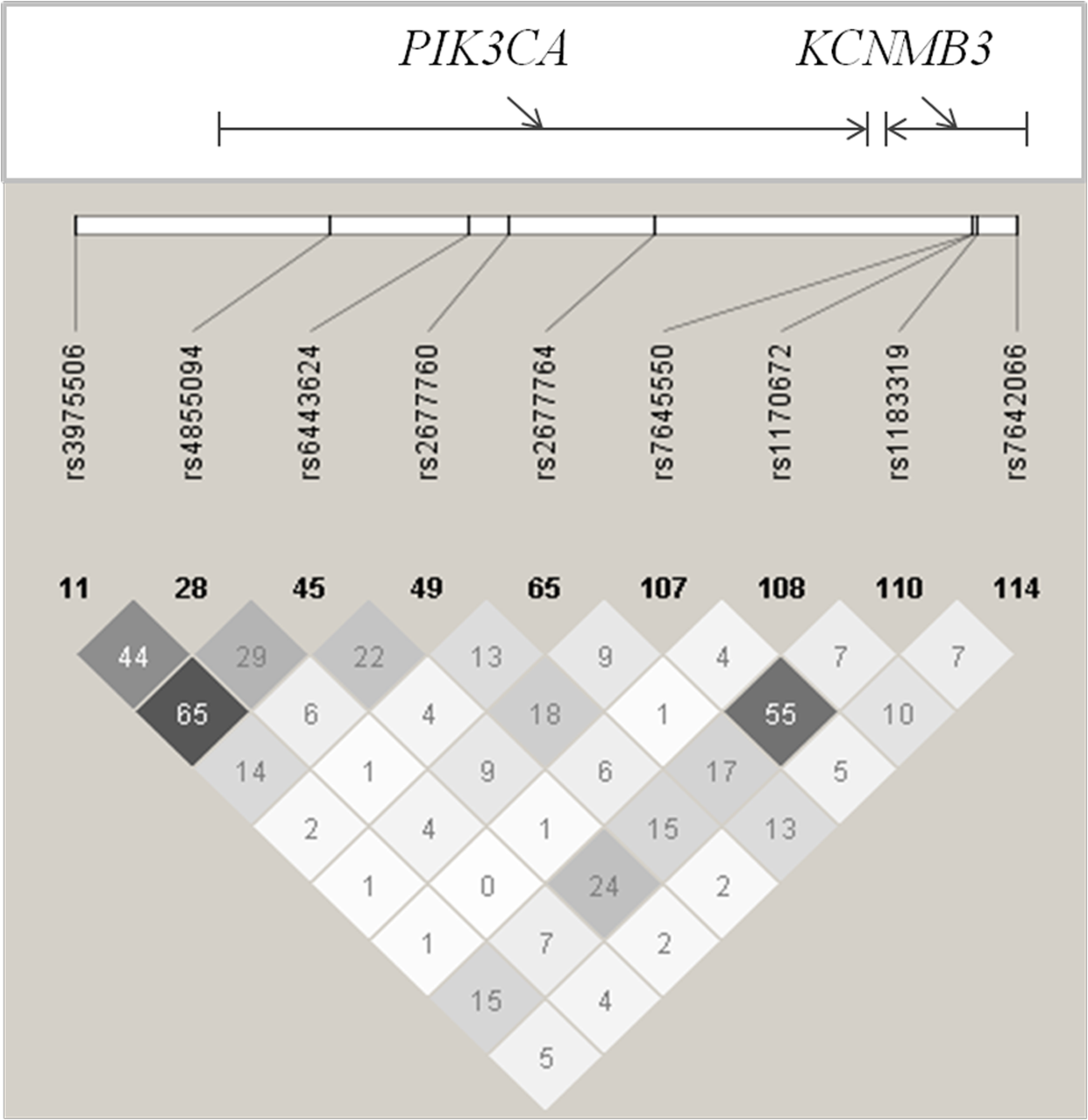

Supplement: Figure S1 — Linkage disequilibrium (LD) plot across PIK3CA-KCNMB3 region in CEU population. The horizontal white bar depicts the 160-kb DNA segment analyzed in the sample. An LD plot is presented at the bottom of the figure: each diamond represents the magnitude of LD for the single pair of markers. The numbers under the variant name and above the plot indicates the order of the corresponding variants among all the variants in the region. The numbers inside the diamonds indicate the r 2 value. (DOCX) [file pone.0067394.s001.docx]
