# Supplementary material for: Polyunsaturated Fatty Acids Modulate the Association between PIK3CA-KCNMB3 Genetic Variants and Insulin Resistance
Source: PLoS One. 2013 Jun 27;8(6):e67394. doi: 10.1371/journal.pone.0067394 (PMC3694924; doi:10.1371/journal.pone.0067394)
Supplement: Table S1 — Description of selected variants in PIK3CA-KCNMB3 region in GOLDN. (DOCX) [file pone.0067394.s002.docx]

**Table S1. Description of selected variants in *PIK3CA-KCNMB3* region in GOLDN^1^**

| SNP Name | Gene | Location | Chromosome position (Genome Build 36.3) | HWE *P*-value | Major/minor allele | MAF | Imputed genotype | MACH r^2^ |
| --- | --- | --- | --- | --- | --- | --- | --- | --- |
| rs3975506 | Intergenic | Upstream PIK3CA | 180324682 | 0.525 | T/C | 0.17 | No |  |
| rs4855094 | PIK3CA | Intron | 180360671 | 0.923 | G/A | 0.07 | No |  |
| rs6443624 | PIK3CA | Intron | 180380368 | 0.693 | C/A | 0.21 | Yes | 0.954 |
| rs2677760 | PIK3CA | Intron | 180385958 | 0.033 | T/C | 0.49 | Yes | 0.952 |
| rs2677764 | PIK3CA | Intron | 180406501 | 0.524 | C/T | 0.12 | No |  |
| rs7645550 | KCNMB3 | Exon-missense | 180451328 | 0.074 | C/T | 0.37 | Yes | 0.924 |
| rs1170672 | KCNMB3 | Exon-missense | 180451354 | 0.15 | T/C | 0.11 | No |  |
| rs1183319 | KCNMB3 | 5’-UTR | 180451801 | 0.893 | A/G | 0.45 | Yes | 0.918 |
| rs7642066 | KCNMB3 | Intron | 180457365 | 0.657 | T/A | 0.44 | Yes | 0.99 |

^1^ HWE *P*-value, Hardy-Weinberg equilibrium expectation *P*-values; MAF, minor allele frequency; MACH r^2^, SNP imputation quality.
