# Supplementary material for: Polyunsaturated Fatty Acids Modulate the Association between PIK3CA-KCNMB3 Genetic Variants and Insulin Resistance
Source: PLoS One. 2013 Jun 27;8(6):e67394. doi: 10.1371/journal.pone.0067394 (PMC3694924; doi:10.1371/journal.pone.0067394)
Supplement: Table S2 — Interactions between dietary PUFA and rs1183319 on fasting glucose and HbA1c in the BPRHS participants. (DOCX) [file pone.0067394.s003.docx]

**Table S2. Interactions between dietary PUFA and rs1183319 on fasting glucose and HbA1c in the BPRHS participants**^1^

| Diet | % energy | Genotypes | Glucose, mmol/L | *P* -trend | *P* -interaction^2^ | HbA1c, % | *P*-trend | *P* –interaction^2^ |
| --- | --- | --- | --- | --- | --- | --- | --- | --- |
| Total PUFA | ≤8.73 | GG + GA (334) | 6.71 ± 0.17 | 0.218 | 0.865 | 7.04 ± 0.10 | 0.162 | 0.882 |
|  |  | AA (80) | 6.31 ± 0.24 |  |  | 6.85 ± 0.19 |  |  |
|  | >8.73 | GG + GA (342) | 6.94 ± 0.15 | 0.278 |  | 7.21 ± 0.10 | 0.322 |  |
|  |  | AA (74) | 7.20 ± 0.36 |  |  | 7.19 ± 0.22 |  |  |
| Total n-3 PUFA | ≤0.68 | AG + GG (346) | 6.63 ± 0.14 | 0.777 | 0.079 | 6.98 ± 0.09 | 0.301 | 0.839 |
|  |  | AA (69) | 6.64 ± 0.27 |  |  | 6.82 ± 0.17 |  |  |
|  | >0.68 | AG + GG (330) | 7.03 ± 0.18 | 0.022 |  | 7.28 ± 0.11 | 0.135 |  |
|  |  | AA (85) | 6.83 ± 0.33 |  |  | 7.17 ± 0.22 |  |  |
| Total n-6 PUFA | ≤8.01 | AG + GG (334) | 6.72 ± 0.17 | 0.236 | 0.945 | 7.04 ± 0.10 | 0.211 | 0.956 |
|  |  | AA (80) | 6.31 ± 0.24 |  |  | 6.85 ± 0.19 |  |  |
|  | >8.01 | AG + GG (342) | 6.93 ± 0.15 | 0.246 |  | 7.21 ± 0.10 | 0.252 |  |
|  |  | AA (74) | 7.20 ± 0.36 |  |  | 7.19 ± 0.22 |  |  |
| n-3: n-6 | ≤0.09 | AG + GG (345) | 6.66 ± 0.13 | 0.733 | 0.301 | 7.02 ± 0.09 | 0.635 | 0.034 |
|  |  | AA (73) | 6.97 ± 0.33 |  |  | 7.15 ± 0.18 |  |  |
|  | >0.09 | AG + GG (331) | 7.00 ± 0.18 | 0.069 |  | 7.24 ± 0.11 | 0.011 |  |
|  |  | AA (81) | 6.54 ± 0.28 |  |  | 6.89 ± 0.23 |  |  |

^1^ Values are mean ± SEM.

^2^ *P*-values were derived from a multivariate interaction model, after adjustment for age, sex, waist circumference, alcohol drinking, smoking status, hormone replacement therapy, type 2 diabetes and population admixture.
