# Supplementary material for: Polyunsaturated Fatty Acids Modulate the Association between PIK3CA-KCNMB3 Genetic Variants and Insulin Resistance
Source: PLoS One. 2013 Jun 27;8(6):e67394. doi: 10.1371/journal.pone.0067394 (PMC3694924; doi:10.1371/journal.pone.0067394)
Supplement: Table S3 — Variance contributions of PIK3CA-KCNMB3 variants and their interaction with dietary n-3: n-6 PUFA ratio for HOMA-IR in GOLDN. (DOCX) [file pone.0067394.s004.docx]

**Table S3. Variance contributions of *PIK3CA-KCNMB3* variants and their interaction with dietary n-3: n-6 PUFA ratio for HOMA-IR in GOLDN^1^**

| SNPs | Main genetic effect on HOMA-IR, % | Interaction with dietary n-3: n-6 PUFA ratio on HOMA-IR, % |
| --- | --- | --- |
| rs3975506 | 0.30 | 0.14 |
| rs4855094 | 0.03 | <0.01 |
| rs6443624 | 0.30 | 0.07 |
| rs2677760 | 1.14 | 0.39 |
| rs2677764 | 0.04 | 0.03 |
| rs7645550 | 0.41 | 0.95 |
| rs1170672 | 0.19 | <0.01 |
| rs1183319 | 0.25 | 1.02 |
| rs7642066 | <0.01 | 0.19 |

^1^ R software (version 2.15.0) GWAF package was used in the estimation of variance contribution. Among these nine SNPs, six independent SNPs (rs3975506, rs2677760, rs2677764, rs1170672, rs1183319, and rs7642066), with r^2^ < 0.20, were selected to estimate the total portion of HOMA-IR variation explained by the *PIK3CA-KCNMB3* variants and their interactions with diet. Total portion of HOMA-IR variation explained by these six independent SNPs was 1.91%, and explained by the interaction of these SNPs with dietary n-3: n-6 PUFA ratio was 1.78%. The statistical model was adjusted for age, sex, waist circumference, alcohol drinking, smoking status, physical activity, type 2 diabetes, study center and family relationships.
